# Artifact subspace reconstruction: a candidate for a dream solution for EEG studies, sleep or awake

**DOI:** 10.1093/sleep/zsad241

**Published:** 2023-09-16

**Authors:** Makoto Miyakoshi

**Affiliations:** Division of Child and Adolescent Psychiatry, Cincinnati Children’s Hospital Medical Center, Cincinnati, OH, USA; Department of Psychiatry, University of Cincinnati College of Medicine, Cincinnati, OH, USA

Traditional sleep EEG applications for identifying sleep stages may require minimal signal processing because well-trained expert eyes can distinguish signals from noise effortlessly. However, basic research on sleep using a high-density EEG system requires signal preprocessing. Manually cleaning these data, 10–20 times longer than recordings from typical cognitive or clinical studies, is labor intensive and not reproducible. The work of Somervail et al. [1] published in this volume demonstrates an elegant solution to this problem. The authors customized the fully automated continuous data cleaning solution called *artifact subspace reconstruction* (ASR) [2–6]. In this brief commentary, I will give a historical and institutional background of how ASR came to be, some general facts, and usage tips.

ASR was developed by Christian Kothe as a part of BCILAB [2] for online signal cleaning within a few hundred-millisecond delay. At that time, I was looking for a solution to clean EEG data recorded from children with chronic tic disorder [7], so I consulted him. He quickly adapted the original code to an offline version upon my request. He made a minor change in the preemphasis filter part to address my claim that the lower end of the PSD of the cleaned data were somewhat unnatural. After receiving the updated offline version, I wrapped it up into an EEGLAB plugin [8] for broader utility by other users. I named the plugin *clean_rawdata*(). Although this name was less poetic than *Dusk2Dawn,* it became the most downloaded plugin in a few years. I maintained the plugin up to version 1.10 while staying true to the original algorithm, before handing over its management to the EEGLAB developers in 2017 upon request.

Roughly speaking, ASR operates as a sliding-window channel interpolation algorithm. Unlike traditional manual data cleaning, ASR does not reject a time window because of outlier values in one of the channels. Instead, it identifies a sparse subset of the contaminated channels and interpolates them using the non-contaminated channels. In EEG, spline interpolation applied to electrode locations is a popular solution [9, 10], but ASR takes different approach; ASR uses principal component analysis to identify the sparse subset of contaminated channels for every sliding window separately. The spatial spread represented by each PC can be interpreted as the effect of volume conduction. To determine the rejection threshold, ASR samples artifact-free parts of the input data as *reference data* in the initial stage to calculate the standard deviation (SD). Users specify the rejection criterion expressed as a multiple of the calculated *SD*. The recommended value is within the range of 10–30 *SD*s [4, 6, 11]. Somervail et al. state that ASR is not merely an adaptive sliding-window principal component rejection algorithm. To explain it, a minimal use of linear algebra description that shows the *core algorithm* of ASR may be helpful.


$$\begin{array}{l} X=MS \end{array}$$

$$\begin{array}{l} Y=V^{T}MS \end{array}$$

$$\begin{array}{l} S={\left(V^{T}M\right)}^{T}Y \end{array}$$

$$\begin{array}{l} S_{clean}={\left(V^{T}M\right)}_{trunc}^{+}Y \end{array}$$

$$\begin{array}{l} X_{clean}=MS_{clean} \end{array}$$

where X is the channel × time matrix of observed signals, M is ASR’s mixing matrix, which is the square root of covariance matrix of reference data, S is a channel × time matrix of ASR’s source signals (note that this S is never explicitly reconstructed), Y is a component × time matrix of the principal component analysis-decomposed X, “clean” represents outlier-free signals, “trunc” represents truncated (PC-rejected) to remove outlier components, and + represents the Moore-Penrose pseudo-inverse. The fully detailed description can be found in [4]. Note $X_{clean}$ is not directly a linear mixing from PC time series Y, but $S_{clean}$ is mixed by M to reconstruct $X_{clean}$.

The optimum cutoff value for the multiplier of SD has been repeatedly studied [4, 6, 11–13]. However, ASR has 30 other parameters to tweak, most of which have never been discussed. Somervail et al. examined the effect of the length of the sliding window and determined to adopt 3–6 times longer window than the default setting. This is because the sleep slow waves they target in the sleep study have a longer time constant than the default value, which is an excellent example of target-guided parameter selection in setting ASR. They also tested a range of parameters for the final selection, as shown in Supplementary Figure S1 of Somervail et al. [1], which is a good practice. Unfortunately, choosing one value in parameter settings is common, often based on qualitative information (e.g. A is good, B is wrong, etc.) without quantitatively evaluating the differences in the results. Considering the parametric changes in the parameter in question is helpful for optimization and for analysts to gain a sense of input–output relations, such as linearity and scale sensitivity.

One might wonder why the previous studies repeatedly recommended abnormally high *SD* = 10–30. These numbers translate to *p* = 10^−23 and *p* = 10^−196, respectively, given a normal distribution. Why do we need to use such extreme values? This question has never been asked. This may be an example of *normalcy bias* i.e. the initial response to a disaster warning is disbelief and people do not take any action [14]. But let us remember the spirit of *doute méthodique* (“methodical doubt”) and recognize the high *SD*s as a problem. I will provide a qualitative clarification below. The issue arises from a part of the *non-core algorithm* of ASR: (1) By default, a maximum of 7.5% of channels are allowed to have outlier values. This is to increase the number of available samples while taking care of outliers in the subsequent robust statistics, (2) When fitting a Gaussian model, ASR uses an asymmetrically truncated Gaussian to ignore the large portion of the right-hand side of the distribution. This is a rather unique trick of ASR for robustification. However, it causes a skew to the right (i.e. the peak shifts to the left) due to the intentionally allowed presence of outliers, and practically only the left half of the original distribution is used to fit a Gaussian model. Consequently, the estimated *SD*s tend to be much smaller than that for the original distribution with a long tail on the right-hand side. This is the (at least one) mechanism of inflation of the *SD*. This issue does not seem to be easily fixable because it derives from the basic design philosophy of the original ASR algorithm. However, the above explanation also implies that although the cause of the bias is systematic, it is not necessarily pathological. Probably, current ASR users implicitly know that ASR’s *SD* for the cutoff is not incommensurable with *SD* in the common-sense statistics. The above explanation provides support for this empirical understanding. The complete details of this argument will be published elsewhere.

The year 2023 marks the tenth anniversary of ASR since the young talented engineer developed ASR single-handedly (he also developed the Lab Streaming Layer around the same time). ASR stands as one of the frontrunner candidates for a reliable, automated, and adaptive EEG cleaning solution—a dream solution for all EEG researchers. But ASR is not a finished project at this point. For example, using the methodical doubt again, ASR’s implicit premise that “ASR is better than mere sliding-window adaptive PC rejection because ASR uses M ” is actually an unvalidated claim. Another important future task is to evaluate frequency dependency of the spatial filter in ASR: for example, beta and gamma band EEG power generally *increases*, not decreases, as a result of spatial-filter component rejection. This is because an output from a spatial filter is dominated by a frequency band with dominant power, and EEG data generally have 1/f power distribution. Thus, a spatial filter works effectively only for low-frequency signals. High-frequency signals are poorly decomposed and remain correlated. Rejecting correlated components causes de-cancelation which introduces the counterintuitive signal power increase. I reported it for the cases of ASR and independent component analysis [15]. The problem will be discussed elsewhere in more detail. Thus, more studies are needed to better establish ASR. Our community needs more multidisciplinary researchers who can discern unique computational problems in different aspects of EEG research and underpin them in the design of simulation studies and mathematical descriptions. Somervail et al. work embodies this spirit, and I join them, hoping their contribution gains wide recognition not only in the *Sleep* community but across all dawn-to-dusk awake communities as well.
